# Insular and Hippocampal Connectivity Is Associated With Perceived Financial Exploitation in Older Adults

**DOI:** 10.1093/geroni/igaa057.1662

**Published:** 2020-12-16

**Authors:** Gali Weissberger, Laura Mosqueda, Annie Nguyen, Jenna Axelrod, Patricia Boyle, Nathan Spreng, Duke Han

**Affiliations:** 1 University of Southern California, Alhambra, California, United States; 2 Keck School of Medicine of the University of Southern California, Los Angeles, California, United States; 3 Rush University Medical College, Chicago, Illinois, United States; 4 McGill University, Montreal, Quebec, Canada

## Abstract

Little is known about the neural correlates of financial exploitation (FE) in older adults. Cognitively-intact older adults who self-reported a history of FE (N=19; M age=69.84, SD=13.06) and demographically-matched non-FE older adults (N=16; M age=65.13, SD=8.48) underwent resting-state fMRI. Predefined regions of interest were prescribed using the Harvard-Oxford atlas for their involvement in tasks of economic decision making: insula, hippocampus, and the medial prefrontal cortex (mPFC). Analyses adjusted for age, education, sex, and MoCA scores; groups did not differ on these factors. Clusters were FDR-corrected with a threshold of p<0.05 (voxel threshold p<0.005), two-tailed. Compared to the non-FE group, the FE group exhibited greater functional connectivity (FC) between the right insula and left temporal lobe regions (t(29)= -4.81), and between the left insula and right temporal lobe regions (t(29)= -5.78). The FE group showed less FC between the left insula and two clusters in the right lateral occipital cortex (t(29)= 5.18) and left cerebellum (t(29)= 4.68). Additionally, FE was associated with greater FC between the right hippocampus and five clusters spanning the right temporal lobe, parietal lobe, and frontal pole (ts(29)= -4.11 to -4.51), and less FC between the right hippocampus and three clusters spanning the bilateral caudate and the left intracalcarine cortex (ts(29)= 4.76-6.03). Groups did not differ in FC patterns with the mPFC. Results suggest that FE is associated with whole-brain FC differences involving the insula and hippocampus among cognitively-intact older adults.

